# Emerging scale invariance in a model of turbulence of vortices and waves

**DOI:** 10.1098/rsta.2021.0080

**Published:** 2022-03-07

**Authors:** M. Shavit, N. Vladimirova, G. Falkovich

**Affiliations:** ^1^ Weizmann Institute of Science, Rehovot 76100, Israel; ^2^ Brown University, Providence, RI 02912, USA; ^3^ Landau Institute for Theoretical Physics, Moscow Region 142432, Russia

**Keywords:** turbulence, entropy, symmetry

## Abstract

This note is devoted to broken and emerging scale invariance of turbulence. Pumping breaks the symmetry: the statistics of every mode explicitly depend on the distance from the pumping. And yet the ratios of mode amplitudes, called Kolmogorov multipliers, are known to approach scale-invariant statistics away from the pumping. This emergent scale invariance deserves an explanation and a detailed study. We put forward the hypothesis that the invariance of multipliers is due to an extreme non-locality of their interactions (similar to the appearance of mean-field properties in the thermodynamic limit for systems with long-range interaction). We analyse this phenomenon in a family of models that connects two very different classes of systems: resonantly interacting waves and wave-free incompressible flows. The connection is algebraic and turns into an identity for properly discretized models. We show that this family provides a unique opportunity for an analytic (perturbative) study of emerging scale invariance in a system with strong interactions.

This article is part of the theme issue ‘Scaling the turbulence edifice (part 1)’.

## 1. Introduction

We owe to Uriel Frisch that scale invariance and its breakdown came into focus for physicists and mathematicians working on turbulence [1]. Pumping and dissipation break scale invariance and time reversibility. Time irreversibility is manifested in the explicit breakdown of the detailed balance and in non-zero energy flux, which is scale-independent [2]. As far as scale invariance is concerned, statistics of the velocity gets independent of the (long) distance to the dissipation scale, but changes with the distance to the pumping scale no matter how large this distance is. Understanding this phenomenon required introduction of simple yet non-trivial models [3] going back to Kolmogorov and developed by Obukhov, Frisch, Kraichnan and others. With the solvable Kraichnan model of passive scalar, turbulence theory got its Ising-model moment [4], when we understood the role of space in keeping scale invariance broken; every scalar’s moment is determined by its own geometrical statistical conservation law. Empirically, the distinction between direct and inverse cascades was noticed: the former had an anomalous scaling, while the latter had not [5,6]. Another aspect of the symmetry breakdown was related to the fractal distribution of the dissipation in space [1]. An alternative approach has been provided by considering one-dimensional chains of interacting modes [7] where there is essentially no space. For such chains, the mode number serves as a logarithm of the effective wavenumber, so that multiplying scales corresponds to adding mode numbers and scale invariance is equivalent to the translation invariance along the chain. Yet, even for the chains with local interactions, called shell models, the breakdown of translation invariance along the chain has been observed [8]. Our paper is devoted to such models and the role of temporal correlations in setting the cascade and its symmetries, broken and emerging. In particular, we elucidate the necessary condition for the breakdown of scale invariance: it requires a cascade to proceed from slow to fast modes; the remarkable illustration of that are inverse cascades with an anomalous scaling.

The above developments lead us to believe that the breakdown, or rather non-restoration, of scale invariance in a turbulent cascade is a natural phenomenon that no longer deserves our astonishment. What we believe is presently much more intriguing is the long-known *emergence* of scale invariance of the so-called Kolmogorov multipliers, which are ratios of the amplitudes of different modes or ratios of the velocity increments taken at different scales [9–15]. In other words, the main question is why the turbulent cascades are multiplicative. Our viewpoint argued below is that precisely because the equations on amplitudes are scale invariant, while the equations on multipliers are not, the statistics of the latter saturates while the statistics of the former does not.

Here, we address the broken and emerging scale invariance of turbulent cascades in a class of systems capable to model two distinct classes of physical phenomena. The first class of phenomena belong to incompressible hydrodynamics, described by the celebrated hydrodynamic Euler equation and many equations for geophysical, astrophysical and magnetohydrodynamic flows. The second class contains systems of resonantly interacting waves. The discretized models of the first class exactly correspond to the second one, as was noticed in [16]. We shall consider one family of such models and describe far-from-equilibrium (turbulent) states of such systems.

## 2. Models

In 1966 V. Arnold noticed the group-theoretic analogy (whose big time in turbulence research may yet to come) between the ordinary differential Euler equations describing solid body dynamics and the partial differential Euler equation describing fluid flows: both correspond to a geodesic equation on the relevant Lie group with respect to a one sided (left or right) invariant metric [17]. On the one hand, the moment of momentum M of a solid body in the rotating reference frame satisfies the Euler equation dM/dt=M×Ω, where $\Omega_{i}=I_{ij}^{-1}M_{j}$ and $I_{ij}$ is the inertia tensor. On the other hand, the velocity curl in an isentropic flow, ω=∇×v, satisfies another equation bearing the name of Euler: ∂ω/∂t=∇×(v×ω). Both Euler equations, for solids and for fluids, have quadratic nonlinearity and possess quadratic invariants. The same is true for the rich family of two-dimensional hydrodynamic models, where a scalar field a (vorticity, temperature, potential) is linearly related to the stream function ψ, which determines the velocity carrying the field: ∂a/∂t=−(v⋅∇)a, v=(∂ψ/∂y,−∂ψ/∂x), $\psi (\text{r})=\int d{\text{r}}^{\prime }|\text{r}-{\text{r}}^{\prime }{|}^{m-2}a({\text{r}}^{\prime })$. For the 2D Euler equation, m=2. Other cases include surface geostrophic (m=1), rotating shallow fluid or magnetized plasma (m=−2), etc. After Fourier transform,

2.1 $$\frac{\partial a_{\text{k}}}{\partial t}=\sum_{\text{q}}\frac{\text{k}\times \text{q}}{q^{m}}a_{\text{q}}a_{\text{k-q}}.$$

In 1969 A. Obukhov suggested to model fluid turbulence by the chains of ODEs having these properties [7]:

2.2 $${\dot{u}}_{i}=\Gamma_{jl}^{i}u_{j}u_{l},\;\Gamma_{il}^{i}=0=\Gamma_{i,jl}+\Gamma_{l,ji}+\Gamma_{j,li}.$$

The indices in (2.2) are lowered using the metric defined by the quadratic invariant $2E=g_{ij}u^{i}u^{j}$. The position of the indices is irrelevant in what follows.

Here, we add a class of models describing resonantly interacting waves with the following Hamiltonian,

2.3 $$H_{w}=\sum_{i}\omega_{i}|b_{i}{|}^{2}+\sum_{ijl}(V_{l,ij}b_{i}^{*}b_{j}^{*}b_{l}+V_{l,ij}^{*}b_{i}b_{j}b_{l}^{*}),$$

where $V_{l,ij}\neq 0$ only if $\omega_{i}+\omega_{j}=\omega_{l}$. By the gauge transformation, $a_{i}=b_{i}\exp(ı\omega_{i}t)$, we can turn the equations of motion, $ı{\dot{b}}_{i}=\partial H_{w}/\partial b_{i}^{*}$ into a system of the type (2.1) and (2.2):

2.4 $$ı{\dot{a}}_{i}=\sum_{jl}(V_{i,jl}^{*}a_{j}a_{l}+2V_{l,ij}a_{j}^{*}a_{l}).$$

The equation (2.4) is Hamiltonian, that is $ı{\dot{a}}_{i}=\partial K/\partial a_{i}^{*}$, where

2.5 $$K=\sum_{ijk}K_{ijk}=\sum_{ijk}(V_{k,ij}a_{i}^{*}a_{j}^{*}a_{k}+V_{k,ij}^{*}a_{i}a_{j}a_{k}^{*}).$$

It conserves not only the Hamiltonian K but also $N=\sum_{i}\omega_{i}|a_{i}{|}^{2}$:

2.6 $$\begin{array}{rl} \frac{\text{d}N}{\text{d}t} & \;=ı\sum_{ijk}\omega_{i}[(V_{i,jk}a_{i}a_{j}^{*}a_{k}^{*}-V_{i,jk}^{*}a_{i}^{*}a_{j}a_{k})+2(V_{k,ij}^{*}a_{i}a_{j}a_{k}^{*}-V_{k,ij}a_{i}^{*}a_{j}^{*}a_{k})]\\ & \;=ı\sum_{ijk}(\omega_{i}+\omega_{j}-\omega_{k})(V_{k,ij}^{*}a_{i}a_{j}a_{k}^{*}-V_{k,ij}a_{i}^{*}a_{j}^{*}a_{k})=0. \end{array}$$

Owing to the extra invariant, two-mode and three-mode systems are integrable.

We consider a sub-class of the Hamiltonian models (2.1) and (2.2) where interactions with only one type of triads in (2.5) are retained (see [18] for a general classification of such models)

2.7 $$H_{A}=\sum_{i}A_{i}(a_{i}^{*}a_{i+p}^{*}a_{i+q}+a_{i}a_{i+p}a_{i+q}^{*}).$$

Here, q>p are non-negative integers. The equations of motion $ı{\dot{a}}_{i}=\partial H_{A}/\partial a_{i}^{*}$ for arbitrary $A_{i}$ have an extra quadratic invariant,

2.8 $$W=\sum_{i}W_{i}|a_{i}{|}^{2},$$

defined by the recursion relation $W_{i}+W_{i+p}=W_{i+q}$ for all i. In the limit i≫1, we shall focus on the scale-invariant power-law functions $W_{i}\to \lambda^{i}$, where λ satisfies the equation $\lambda^{q}=\lambda^{p}+1$. The first two families have, respectively, p=0,q=1 and p=1,q=2:

2.9 $$H_{U}=\sum_{i}U_{i}(a_{i}^{*2}a_{i+1}+a_{i}^{2}a_{i+1}^{*})$$

and

2.10 $$H_{V}=\sum_{i}V_{i}(a_{i}^{*}a_{i+1}^{*}a_{i+2}+a_{i}a_{i+1}a_{i+2}^{*}).$$

The models can be used in numerous classical and quantum applications, since i can denote real-space sites, spectral modes, masses of particles, number of monomers in a polymers, etc. Gauge invariance $a_{m}\to a_{m}\;{\text{e}}^{ıW_{m}t}$ means, in particular, that (2.7) describes resonant interaction of waves with frequencies $W_{i}$. Apart from decay and coalescence of waves or quantum particles, the Hamiltonian also describes breakdown and coagulation of particles or polymerization of polymers. In particular, the U-model has λ=2 and describes the chain of frequency-doubling interactions, which can be realized by a cascade of nonlinear elements (e.g. lasers) each resonantly generating the second harmonic. Respectively, the V-model describes a resonant interaction of waves whose frequencies relate as the Fibonacci numbers $W_{i}=F_{i}=\{1,1,2,3,5\ldots \}$ defined by the identity $F_{i}+F_{i+1}=F_{i+2}$ with $F_{0}=0$ and $F_{1}=1$ . In this case $W_{i}=F_{i}=[\phi^{i}-{\left(-\phi \right)}^{-i}]/\sqrt{5}$, so that $\lambda =\phi =(1+\sqrt{5})/2$ is the golden mean [16]. Such models of three-mode interaction, in particular, are used to describe ubiquitous Fibonacci patterns in plant leaves, pinecones, sunflower seeds, cactus spines, pineapple scales, etc. [19,20]. If i are spectral parameters, they are usually understood as shell numbers with frequencies/wave numbers being $W_{i}=\lambda^{i}$, so that power-law dependencies on $W_{i}$ mean exponential dependencies on i. The family (2.7) thus belongs to the aforementioned class of shell models [8], that is (2.2) with neighbouring interactions.

Class of models (2.1)–(2.3) is ideally suited for elucidating the role of temporal correlations in the persistence of breakdown of scale invariance of amplitudes and emerging scale invariance of Kolmogorov multipliers. In addition, it allows one to address the question of the cascade direction, which goes back to the paper of Fournier & Frisch [21].

## 3. Thermal equilibrium

In a closed general system (2.3), the microcanonical equilibrium $P=\delta (K-K_{0})\delta (N-N_{0})$ generally has both $N_{0},K_{0}$ non-zero.

To describe the canonical equilibrium and then turbulence, we add white-in-time pumping and dissipation to the general system (2.4)

3.1 $${\dot{b}}_{i}=-\frac{ı\partial K}{\partial b_{i}^{*}}+\xi_{i}-\gamma_{i}b_{i}.$$

Here $\langle \xi_{i}b_{i}^{*}\rangle =P_{i}/2$. Then it is straightforward to show that $\sum_{ijk}\text{d}\langle K_{ijk}\rangle /\text{d}t=-\sum_{ijk}(\gamma_{i}+\gamma_{j}+\gamma_{k})\langle K_{ijk}\rangle$, so that in a steady state $\sum_{ijk}(\gamma_{i}+\gamma_{j}+\gamma_{k})\langle K_{ijk}\rangle =0$. We also have $\text{d}\langle N\rangle /\text{d}t=\sum_{i}(\omega_{i}P_{i}-2\gamma_{i}n_{i})=0$. If pumping and damping are in a detailed balance, that is for every i we have $\omega_{i}P_{i}/\gamma_{i}=T$, then the Gaussian distribution

3.2 $$P_{0}\{b_{i}\}=Z^{-1}\exp\left(-\frac{N}{T}\right)=Z^{-1}\exp(-\sum_{i}\frac{\omega_{i}|a_{i}{|}^{2}}{T}),$$

is the solution to the Fokker–Planck equation

$$\partial_{t}P_{0}=\{K,P_{0}\}=ı\sum_{ijk}(V_{ijk}a_{i}a_{j}a_{k}^{*}-V_{ijk}a_{i}^{*}a_{j}^{*}a_{k})(\omega_{i}+\omega_{j}-\omega_{k})P_{0}=0.$$

The distribution (3.2) realizes the entropy maximum under conditions of fixed finite ⟨N⟩ and ⟨K⟩=0, because any non-zero $\left\langle K_{ijk}\right\rangle$ diminishes entropy. Let us stress that the equilibrium distribution is neither of the naive Gibbs distributions, exp⁡(−K/T) or exp⁡(−H/T), which are both non-normalizable. By contrast, the distribution (3.2) is normalized. As in the general case (2.4) and independent of $A_{i}$, thermal equilibrium in the sub-class (2.5) has exactly Gaussian distribution of statistically independent modes, despite the system being described by a cubic Hamiltonian and thus strongly interacting. That Gaussian distribution (3.2) can serve as a starting point for a perturbation theory near the case where turbulent cascade is close to thermal equilibrium, as can be seen below.

## 4. Turbulent cascades

In a turbulent cascade, pumping and dissipation act on different modes. In the so-called inertial interval between pumping and dissipation, there exists the flux of the quadratic invariant, which can be expressed via the third cumulant:

4.1 $$J_{i}\equiv \text{Im}\langle a_{i}a_{i+p}a_{i+q}^{*}\rangle$$

and

4.2 $$W_{i}\frac{\text{d}\langle |a_{i}{|}^{2}\rangle }{\text{d}t}=W_{i}(A_{i-q}J_{i-q}-A_{i-p}J_{i-p}-A_{i}J_{i})=\Pi (i-1)-\Pi (i)=-\partial_{i}\Pi (i).$$

The right-hand side is the discrete divergence of the flux

4.3 $$\Pi (m)\equiv -\sum_{i=M+1}^{m}W_{i}\frac{\text{d}\langle |a_{i}{|}^{2}\rangle }{\text{d}t}=\sum_{k=m+1-q}^{m-p}2W_{k+q}A_{k}J_{k}+\sum_{k=m-p+1}^{m}2W_{k}A_{k}J_{k}+\Pi_{M},$$

where $\Pi_{M}$ is a constant independent of m, and M is the beginning of the inertial interval, i.e we assume that (2.4) is valid for i>M. The third-order cumulants are zero in equilibrium, but in turbulence they are non-zero to carry the flux. In a steady turbulence, the cumulant is as follows:

4.4 $$J_{m}=\frac{CW_{M+p-q-m}}{A_{m}},$$

where C depends on p,q but not on k. Indeed, it is straightforward to check that (4.1) turns (4.2) into zero. In particular for the Fibonacci V-model $\Pi (m)=2C_{V}F_{M}$ and for the chain of doubled harmonics, U models, $\Pi (m)=C_{U}2^{M+1}$.

For i≫1, it is natural to assume that both Hamiltonian coefficients turn into power laws:

4.5 $$W_{i}=\lambda^{i}\;\text{and}\;A_{i}=\lambda^{i\alpha }.$$

Since we are interested in scale invariance, in what follows we shall assume (4.5) to be valid for all i. Then the third cumulant (4.4) is a power law too with the scaling exponent determined by the flux constancy

4.6 $$J_{m}\propto \lambda^{-m(1+\alpha )}.$$

Gauge invariance requires that the triple cumulants other than (4.1) are zero and that the pair correlation function is diagonal in any state: $\langle a_{m}a_{n}^{*}\rangle \propto \delta_{mn}$ (when modes are spatial harmonics, diagonal pair correlation function comes from translation invariance in space). This does not mean that different modes are uncorrelated in turbulence. The pair correlation function is a proper measure of correlation only for Gaussian statistics. Below, we introduce the universal measure of two-mode correlation—the mutual information—and show that it is non-zero in turbulence.

## 5. Cascades and their directions

Which direction does the cascade (4.6) go? Frisch & Fournier argued that the stationary turbulence spectrum is to be formed if its time derivative is a growing function of its slope [21,22]. Indeed, consider the density of some conserved quantity $\epsilon_{k}$, which satisfies the equation $\partial_{t}\epsilon_{k}=I_{k}=-\partial_{k}\Pi$ that has a stationary solution $\epsilon_{k}=k^{-s_{0}}$ with a constant flux Π=const. Imagine, for instance, that the stationary direct cascade has been formed between the pumping and some wavenumber k. The spectrum will propagate further into the region of larger k, where $\epsilon_{k}$ is smaller and s is larger, if $I_{k}$ is positive there: $I_{k}(s)\propto s-s_{0}$ or ${\left(\text{d}I/\text{d}s\right)}_{s_{0}}>0$. Similar argument can be made for an inverse cascade, where the condition for propagation is opposite: ${\left(\text{d}I/\text{d}s\right)}_{s_{0}}<0$. On power spectra $\epsilon_{k}=k^{-s}$, we must have the power law, $I_{k}(s)\propto k^{b(s-s_{0})-1}I(s)$, where b is some positive number (b=2 for weak turbulence [22]). That gives the flux $\Pi (s,k)\propto I(s)k^{b(s-s_{0})-1}/b(s-s_{0})$, which turns into a k-independent constant at $s=s_{0}$: $\Pi (s_{0},k)\propto {\left(\text{d}I/\text{d}s\right)}_{s_{0}}$. The last formula means that the flux direction determines the propagation direction, if such reasoning is correct. For a single conserved quantity, the flux sign coincides with that of $s_{0}$, since any distribution steeper than equipartition must carry a positive flux. While it seemed natural that the stationary spectrum must start forming from pumping and propagate into the direction of its flux, the studies of weak wave turbulence established that the ultimate formation of the cascade spectrum does not necessarily proceed from pumping to damping [23,24]. Instead, it proceeds in the direction of deceleration, from fast to slow modes, according to the information-theory argument [25].

The principal difficulty in applying the Frisch–Fournier criterion to systems with a strong interaction is that the flux constancy does not anchor the spectrum, but some higher-order moment, like the third moment for incompressible turbulence and for our system. In our case though, there is a way to bypass this (closure) problem and get an analytic insight into turbulence properties. To do that, we exploit an extra symmetry $a\to -a^{*}$, which provides the existence of an invariant sub-space of solutions with purely imaginary $a_{k}=i\rho_{k}$ for all k

5.1 $$\frac{\partial \rho_{i}}{\partial t}=A_{i-q}\rho_{i-q+p}\rho_{i-q}-A_{i-p}\rho_{i-p}\rho_{i-p+q}-A_{i}\rho_{i+p}\rho_{i+q}.$$

The dynamical equation (5.1) has a stationary solution

5.2 $$\rho_{m}=\lambda^{-m(1+\alpha )/3}.$$

It turns (5.1) into zero everywhere except the chain ends, where the last term is uncompensated at the left end, and the first term at the right end. To compensate these terms and make a true steady state one needs to add pumping and damping at the opposite ends of any finite chain. Therefore, that stationary solution carries a flux—indeed, (5.2) corresponds to (4.6). Each such solution can describe either direct or inverse cascade, since the symmetry ρ→−ρ, t→−t means that one reverses the flux by changing the sign of ρ. The sign of the flux must be determined by our only parameter α, which controls how the interaction strength depends on the mode number. We can now establish the direction of the flux comparing the flux solution with the equipartition $\rho_{m}^{2}\propto \lambda^{-m}$—note that this is *not* the steady solution of the dynamical equation, it requires contact with a thermostat that adds pumping and damping acting on every mode. The flux distribution coincides with equipartition for α=1/2. Physical common sense suggests that the cascade must carry the conserved quantity $\sum_{m}\lambda^{m}\rho_{m}^{2}$ from excess to scarcity [22]. For α>1/2, the steady solutions must correspond to a direct cascade, since they decay with m faster than the equipartition. By the same token, we must have an inverse cascade for α<1/2. These conclusions were supported for V-model at α=1 and α=0, and a double-cascade turbulence was observed at α=1/2 in our previous work [16]. The simplest degree of non-Gaussianity is the dimensionless third cumulant called skewness:

$$\xi =J_{i}/n_{i}^{3/2}.$$

For both cascades at α=1/2, the skewness is of order unity at the damping region and decays towards inertial interval as the inverse distance [16]. The longer the interval, the smaller is ξ at a given distance from the pumping region. This suggests that the limit of long intervals may then be amenable to an analytical treatment, since the statistics is close to equilibrium Gaussian.

One can also write a complex steady solution of the dynamic equation. For U-model it takes the form: $a_{m}=\lambda^{-m(1+\alpha )/3}\;{\text{e}}^{ı\varphi_{m}}$, where the phases are given by the recursive relation $\varphi_{i+1}=2\varphi_{i}\pm \pi /2$, where the sign depends on the cascade direction.

**Figure 1.**
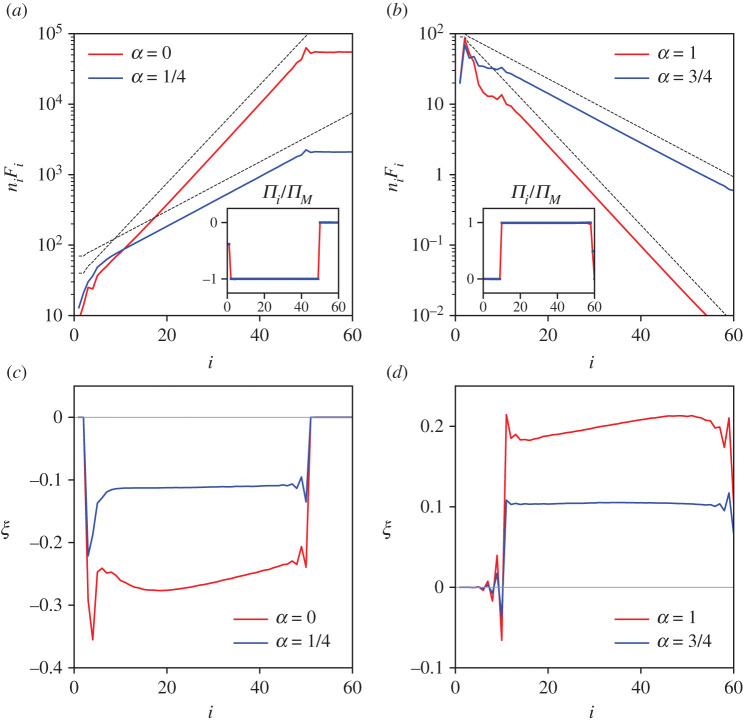
Compensated spectra, fluxes and skewness for α<1/2 (*a*,*c*) and α>1/2 (*b*,*d*). All cases correspond to the same input flux, $\Pi_{p}=67.65$. The pumping is applied to mode p=50 for α<1/2 and to mode p=10 for α>1/2. On the no-flux sides damping is γ=0; on the flux side damping rates are $\gamma_{L}=1.5$ for α=0, $\gamma_{L}=1$ for α=1/4, $\gamma_{R}=28$ for α=3/4, and $\gamma_{R}=3500$ for α=1. The slopes of the dashed lines in the upper panels are 1/3 and 1/6. Approximately, ξ∝(α−1/2).(Online version in colour.)

Figure 1 presents our results for the V-model with α=0,1/4,3/4,1 obtained from the numerical solution ${\dot{a}}_{i}=-ı[V_{i-2}\;a_{i-2}a_{i-1}+V_{i-1}\;a_{i-1}^{*}a_{i+1}+V_{i}\;a_{i+1}^{*}a_{i+2}]-\gamma_{i}a_{i}+\xi_{i}$ with $V_{i}=F_{i}^{\alpha }$. (The way numerics are done is described in [16]). The upper panels show that the occupation numbers approximately follow the power laws $n_{i}\propto \lambda^{-i(1+\alpha )/2}$, which corresponds to (5.2). The lower panel shows how the skewness decreases as α approaches 1/2 from either side. For |α−1/2|=1/2, the skewness increase along the cascade is visible.

The only physics that distinguishes one direction along the chain from another is the growth/decay of the typical inverse interaction time $A_{i}a_{i}≃A_{i}J_{i}^{1/3}\propto \lambda^{i(2\alpha -1)/3}$. Not surprisingly, the same parameter α−1/2 also sets the cascade direction. We conclude that cascades proceed from slow modes to fast modes in our system at α≠0, as well as in shell models [16,26–28]. That leads to strong intermittency and long correlations, as we shall see in the subsequent sections. In the degenerate case α=1/2, there is no exponential dependence of the interaction time on the mode number; if one estimates the inverse interaction time as $A_{i}a_{i}≃A_{i}n_{i}^{1/2}$, then it increases along both cascades by a weaker power law.

## 6. Anomalous scaling and breakdown of translation invariance

**Figure 2.**
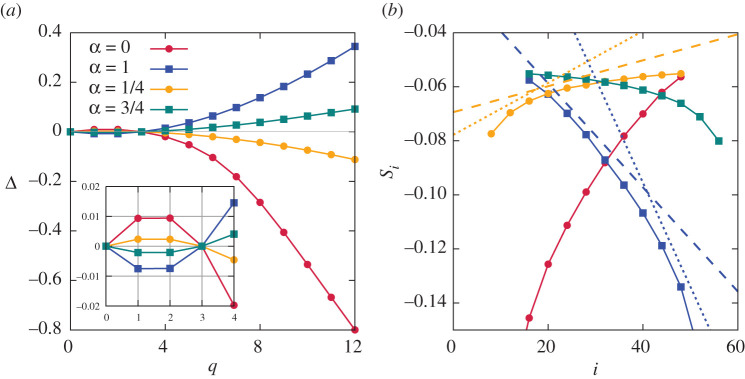
(*a*) Anomalous exponents $\Delta (q)=q\zeta_{3}/3-\zeta_{q}$. (*b*) The one-mode entropy of the complex amplitude normalized by $\sqrt{n_{i}}$. The dashed lines have the slopes Δ(1)ln⁡(ϕ)/2 and the dotted lines Δ(1)ln⁡(ϕ) with the respective Δ(1) taken from *a*. (Online version in colour.)

Apart from skewness, other measures of non-Gaussianity increase along a cascade, as was noticed in [16] for α=0,1. The simplest of such measures is given by the single-mode moments $\langle |a_{i}{|}^{q}\rangle \propto k_{i}^{-\zeta_{q}}=\lambda^{-i\zeta_{q}}$, which in our case are finite for q>−2. The anomalous scaling exponents, $\Delta (q)=q\zeta_{3}/3-\zeta_{q}$, give particular measures of how non-Gaussian cumulants grow along the cascade. They also quantify the breakdown of scale invariance (translation invariance along i in our case). Indeed, all Δ(q)≡0 for any probability distribution $P(a_{i}/J_{i}^{1/3})$ invariant with respect to shifts along i. The flux law $J_{i}\propto \lambda^{-i(1+\alpha )}$ predicts $\zeta_{3}=\alpha +1$; the numerical fit for $\zeta_{3}$ gives 1.0044, 1.2511, 1.7482, 1.9989 for α=0, 1/4, 3/4, 1, respectively, which is a pretty good agreement with the theory. Note the small but measurable anomalous exponent of the second moment, which provides the increase of skewness along the cascade. The anomalous scaling is observable in numerics for all the single-cascade cases presented here, see figure 2*a*. All fits for $\zeta_{q}$ are done for modes 5–25 counting from pumping. Three main conclusions can be drawn from the figure. First, the exponents are nonlinear functions of q, which means breakdown of scale invariance of the one-mode statistics. Second, the high moments deviate stronger from linear scaling, that is the degree of non-Gaussianity increases along the cascade. Third, the closer is α to 1/2 the smaller are the anomalous scaling exponents and respective deviations from Gaussianity.

The most general measure of non-Gaussianity and non-equilibrium is the entropy of the statistical distribution. If we normalize the amplitude by its RMS value, the Gaussian distribution of independent modes realizes the entropy maximum. By the same token, equilibrium distribution corresponds to the entropy maximum at given values of the integrals of motion N,K. Turbulence is an ultimate far-from-equilibrium state, so its statistical distributions must have much lower entropies. Alternatively, one may say that creating turbulence requires processing a lot of information.

That information is encoded in the statistics of the single-mode amplitude and in the inter-mode correlations. Figure 2*b* presents the one-mode entropy decrease along the cascade: $S_{i}=S(x_{i})=S(|a_{i}|/\sqrt{n_{i}})$ is plotted as the function of |i−p|. The entropy can be related to the anomalous exponents using the multi-fractal formalism of Parisi & Frisch [1,29]: the moments $\langle x_{i}^{q}\rangle \propto \lambda^{-i(\zeta_{q}+q\zeta_{2}/2)}$ in the limit of large |i−p| correspond to the multi-fractal distribution,

6.1 $$P(x_{i})\propto \int g\left(\frac{x_{i}}{\lambda^{ih}}\right)x_{i}^{-1}\exp[if(h)\ln\lambda ]\;\text{d}h.$$

Here, g determines the probability distribution of $x_{i}$ at the subset with the given exponent h, while the probability of the subset is determined by $f(h)=\min_{q}(\zeta_{q}-q\zeta_{2}/2-qh)$, that is f(h) is the Legendre transform of ζ(q). The entropy is then

$$S_{i}=-\int \text{d}xP\ln P\propto i[\Delta^{\prime }(0)-\frac{\Delta (2)}{2}]\ln\lambda .$$

This decay is approximately linear in i, as indeed can be seen in figure 2, where i is counted from pumping. Since numerical estimates of the pretty small exponents Δ(1),Δ(2) are approximate, we estimate $\Delta^{\prime }(0)$ as somewhere between two limits. The lower limit is given by Δ(1), which corresponds to the dashed straight lines in figure 2*b*, shown in blue for the direct cascade at α=1 and in yellow for the inverse cascade at α=1/4. To obtain the upper limit, we note that Δ(1)≈Δ(2) and approximate Δ(q) with parabola passing through four points, q=0,1,2,3. That gives the estimate $\Delta^{\prime }(0)=3\Delta (1)/2$, which we take as an upper limit giving the dotted straight lines in figure 2. We see that the respective straight lines give the right order of magnitude for the rate of the entropy decay in the inertial interval both for the direct and inverse cascades. The anomalous exponents for large moments, p≫1, grow twice faster for the inverse cascade: Δ(j)≈−j/16 for α=1 and Δ(j)≈j/8 for α=0. And yet one can see from figures 2 that the one-mode entropy is essentially the same in both cascades. The difference between the cascades in their information content can be seen in the entropies of two-mode distributions, to which we now turn.

**Figure 3.**
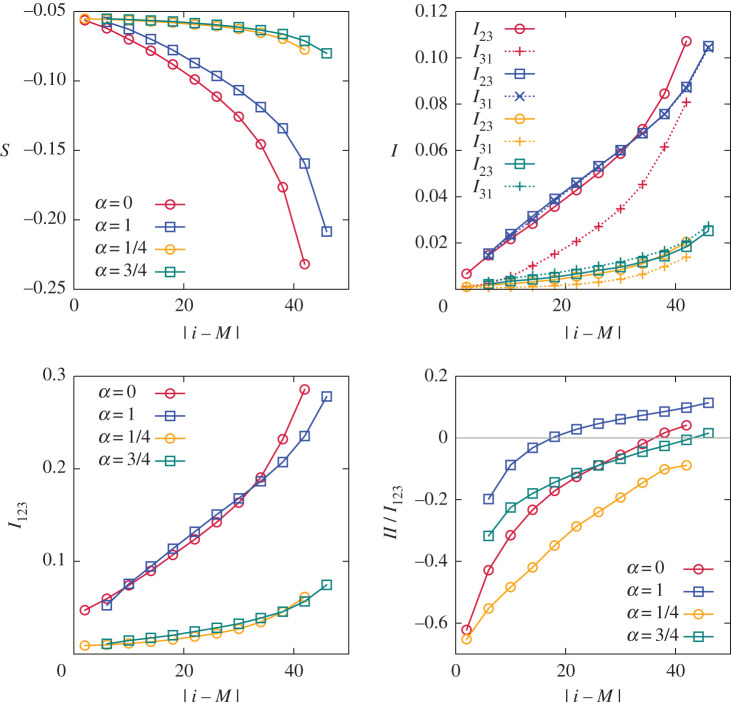
Entropy deviation from equilibrium, the mutual information and the interaction information. All decrease as α approaches 1/2. (Online version in colour.)

The information encoded in the inter-mode correlations is measured by the mutual information between two modes

$$I_{ij}=S(a_{i})+S(a_{j})-S(a_{i},a_{j}),$$

which is the amount of information one can learn about one mode by measuring another. Gauge invariance makes all pair correlation functions between modes identically zero, yet the mutual information is non-zero for any pair in the interacting triplet, as shown in figure 3. We also compute the three-mode mutual information $I(a_{1},a_{2},a_{3})=\sum_{i=1}^{3}S(a_{i})-S(a_{1},a_{2},a_{3})$. Figure 3 shows that the changes along the cascade in one-mode entropy and in two-mode and three-mode mutual information are comparable, that is one obtains comparable amount of information about turbulence from these quantities.

To quantify the influence of one of three variables on the amount of information shared between the other two, one computes what is called interaction information in the classical statistics and topological entanglement entropy in the quantum statistics [30,31]:

6.2 $$\begin{array}{rl} II_{i} & \;=S(a_{i})+S(a_{i+1})+S(a_{i+2})+S(a_{i},a_{i+1},a_{i+2})-S(a_{i},a_{i+1})-S(a_{i},a_{i+2})-S(a_{i+1},a_{i+2})\\ & \;=I_{i,i+1}+I_{i,i+2}+I_{i+1,i+2}-I_{i,i+1,i+2}, \end{array}$$

Positive II(X,Y,Z) measures the redundancy in the information about Y obtained by measuring X and Z separately, while negative one measures synergy which is the extra information about Y received by knowing X and Z together. Note that for a Markov chain, the interaction information is always positive, so that negative II is a reminder that our chain is not Markov. While the entropy itself depends on the units, both the mutual information and the interaction information do not. They are also symmetric, that is they measure the degree of correlation rather than causal relationship or cascade direction. The entropy for an individual mode is presented relative to the Gaussian entropy based on the average occupation number. The details of the numerical procedure of computing entropies and mutual information are described in [16].

## 7. Emerging self-similarity and translation invariance

**Figure 4.**
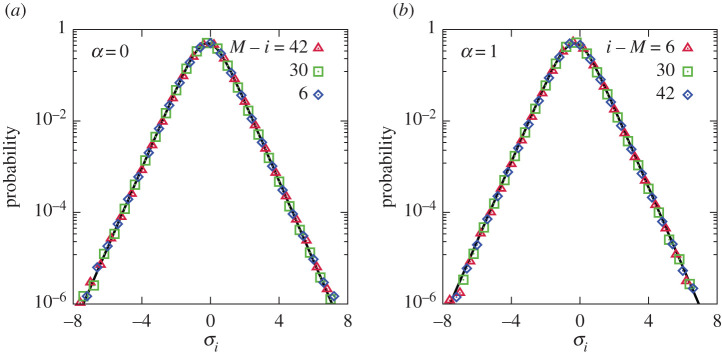
Probability distributions of the Kolmogorov multipliers $\sigma_{i}=\ln|a_{i}/a_{i-1}|$ for different positions in the inverse (*a*) and direct (*b*) turbulent cascades. Solid lines correspond to the thermal equilibrium $P(\sigma )=1/2\cosh^{2}(\sigma -\bar{\sigma })$, where $\bar{\sigma }=-(1/3)\ln\phi$ for the inverse cascade and $\bar{\sigma }=-(2/3)\ln\phi$ for the direct one. The variance $\langle {\left(\sigma -\bar{\sigma }\right)}^{2}\rangle ≃0.75$ for both cascades, which is much larger than either of the squared mean values. (Online version in colour.)

The equations of fluid mechanics without pumping and dissipation are scale-invariant. Similarly, our family of models (2.7) with (4.5) is invariant with respect to translation i→i+c or re-scaling $W\to W\lambda^{c}$, as long as one renormalizes $a_{i}\to a_{i}\lambda^{-c\alpha }$. Turbulence-producing pumping breaks the invariance. Contrary to a general perception, we believe that it is quite natural that turbulent statistics is not scale invariant and depends explicitly on the pumping scale or position. If, however, scale invariance (or even its extension, conformal invariance [32]) emerges far away from the pumping position, *that* needs an explanation. Such emerging scale invariance takes place for the amplitude ratios, called Kolmogorov multipliers, $z_{i}=a_{i}/a_{i-1}$, [3] in shell models and hydrodynamic equations [9–11,14]. Likewise, our numerics show that the statistics of the multipliers is translation invariant as seen from figure 4, which presents the probability distributions for $\sigma_{i}=\ln|z_{i}|$ at different i. We find that the probability distribution of a single multiplier is not only the same for all positions far enough from pumping, but also indistinguishable from the equilibrium distribution,

7.1 $$P_{\text{eq}}(\sigma )=\int \int_{0}^{\infty }\;\text{d}x\;\text{d}y\;{\text{e}}^{-x-y}\delta (\sigma -\frac{1}{2}\ln\frac{x}{y})=\frac{1}{2\cosh^{2}\sigma }.$$

Let us now discuss a hypothetical explanation for such emerging invariance. To avoid unnecessary details in the analytic treatment, we again consider the purely imaginary case $a_{i}=ı\rho_{i}$ and denote $k_{i}=|z_{i}|=\rho_{i}/\rho_{i-1}$. We believe that it is important that the equations on the multipliers are themselves not invariant, in contrast with the equations on the amplitudes. It is this explicit dependence on the position (or scale) which could be responsible for the appearance of the universal position-independent statistics far from the pumping. Namely, the equations on the multipliers have the following form for our models:

7.2 $${\dot{k}}_{i}=A_{i-q-1}\rho_{i-q-1}f(k_{i-q},\ldots ,k_{i+q}).$$

Here, f is a function of a finite number of multipliers, which is translation-covariant. For example, for the U-model

7.3 $${\dot{k}}_{i}=U_{i-2}\rho_{i-2}(2^{\alpha }k_{i-1}-2^{1+2\alpha }k_{i-1}k_{i}^{2}k_{i+1}-k_{i}k_{i-1}^{-1}+2^{1+\alpha }k_{i}^{2}k_{i-1}),$$

while for the V-model at α=0,

$$\begin{array}{rl} {\dot{k}}_{i} & \;=V\rho_{i-3}(k_{i-2}-k_{i-2}k_{i-1}k_{i}^{2}-k_{i-2}k_{i-1}k_{i}^{2}k_{i+1}^{2}k_{i+2}-k_{i-1}^{-1}k_{i}+k_{i-2}k_{i}^{2}+k_{i-2}k_{i-1}k_{i}^{3}k_{i+1}). \end{array}$$

What breaks the covariance is the factor

7.4 $$\rho_{i-q-1}=\rho_{M}\prod_{j=M+1}^{i-q-1}k_{j},$$

which involves all the $k_{j}$ between the pumping position M and the position i−q−1. Here, one can assume $\rho_{M}$ having Gaussian statistics. However, even when i is very far from M, one cannot assume this product statistically independent of the arguments of f. The fundamental reason is that the cascade proceeds from slow to fast modes, so that all the factors in the product (7.4) fluctuate slower than the arguments of f. Let us stress the fundamental difference between the equations on the amplitudes, which are local, and the non-local equations on $k_{i}$, which involve all the multipliers, starting from pumping up to the given mode. While we cannot rigorously prove it yet, we find it plausible that the fact that all i−q−1−M random multipliers in (7.2) and (7.4) are correlated makes correlation between any finite number of them weak, which must be eventually responsible for the emerging translation invariance of the statistics of $k_{i}$ in the limit |i−M|≫1,p,q. The analogy is the validity of the mean-field description in systems with long-range interaction [33]. It is not yet clear how to make a meaningful mean-field approximation in (7.2) and (7.4) to establish explicitly the asymptotic universal statistics of $k_{i}$ in a general case. However, in the degenerate symmetric case α=1/2, the interaction time for the statistically stationary solution is independent of the mode number, and the statistics of the amplitudes is Gaussian. In that case, one may hope to find analytically the joint statistics of the multipliers. Normalized for zero mean and unit variance, we compute for $\sigma_{i}=\ln k_{i}$:

7.5 $$\begin{array}{rl} P_{\text{eq}}(\sigma_{1},\ldots ,\sigma_{n}) & \;=2^{n}n![1+{\text{e}}^{2\sigma_{1}}(1+{\text{e}}^{2\sigma_{2}}(1+\cdots +{\text{e}}^{2\sigma_{n-1}}(1+{\text{e}}^{2\sigma_{n}}){]}^{-n-1}\exp\left[\sum_{k=0}^{n-1}2(k+1)\sigma_{n-k}\right]. \end{array}$$

The case n=1 is (7.1) given above. One could use (7.5) as a starting point for the perturbation theory in small deviations ϵ=α−1/2, which can hopefully demonstrate the universality and translation invariance of the statistics of $k_{i},\sigma_{i}$.

Let us consider the U model. In the dimensionless variables $u_{i}=\rho_{i}2^{i(1+\alpha )/3}$ and $\kappa_{i}=k_{i}2^{(1+\alpha )/3}$, equation (7.3) takes the form

7.6 $${\dot{\kappa }}_{i}=2^{i(2\alpha -1)/3+(2-\alpha )/3}u_{i-2}\kappa_{i-1}(1-\kappa_{i}^{2}\kappa_{i+1}-\kappa_{i}\kappa_{i-1}^{-2}+\kappa_{i}^{2}).$$

For α=1/2, the equations become particularly simple

7.7 $${\dot{u}}_{i}=\sqrt{2}(u_{i-1}^{2}-u_{i}u_{i+1})$$

and

7.8 $${\dot{\kappa }}_{i}=\sqrt{2}{\tilde{\rho }}_{M}(1-\kappa_{i}^{2}\kappa_{i+1}-\kappa_{i}\kappa_{i-1}^{-2}+\kappa_{i}^{2})\prod_{j=M+1}^{i-1}\kappa_{j}\equiv {\tilde{\rho }}_{M}F_{i}(\kappa_{M+1}\ldots \kappa_{i+1}).$$

The goal is to show that the asymptotic invariant measure for that stochastic system is close to the equilibrium measure (7.5), which can be written as

7.9 $$P(\kappa_{1},\ldots ,\kappa_{n})=\frac{2^{n}n!\kappa_{n}\kappa_{n-1}^{3}\ldots \kappa_{1}^{2n-1}}{[1+\kappa_{1}^{2}(1+\kappa_{2}^{2}(1+\cdots +\kappa_{n-1}^{2}(1+\kappa_{n}^{2}){]}^{n+1}}.$$

Treating $\rho_{M}$ as a white noise, we can write the Fokker–Planck equation

7.10 $$\frac{\partial P}{\partial t}\propto \sum_{i,j}\frac{\partial^{2}}{\partial \kappa_{i}\partial \kappa_{j}}F_{i}F_{j}P.$$

where $F_{i}$ is defined via (7.8). The challenge is to check whether (7.9) can be an asymptotic solution of (7.10). One may also wonder if there exists a renormalization group (RG) flow, of which (7.5) and (7.9) is a fixed point.

**Figure 5.**
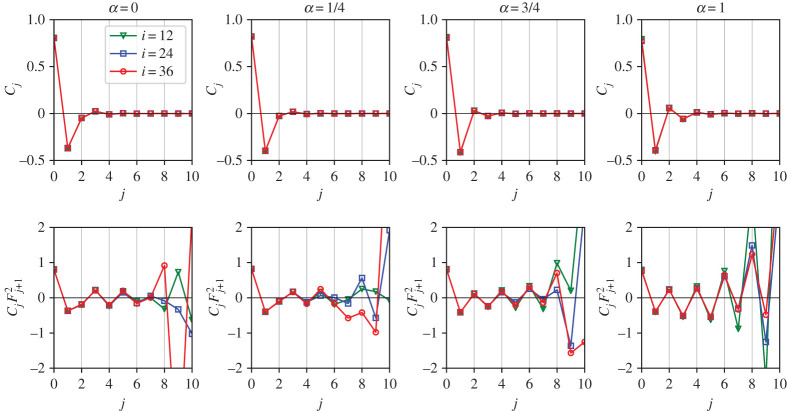
The correlation functions of the logarithms of multipliers $C_{j}=\langle \sigma_{i}\sigma_{i+j}\rangle$ for V-model. From left to right, α is increasing as 0, 1/4, 3/4, 1. Top- original, bottom—multiplied by $F_{j+1}^{2}$ to compensate for exponential decay. (Online version in colour.)

It is important to stress that extreme non-locality of interaction between multipliers, as expressed by the dynamic equation (7.8), does not mean strong correlations between distant multipliers. Figure 5 shows the pair correlation function (compare with [10–13]), which decays exponentially fast with the relative distance for all four values of α. We also computed numerically the mutual information between the neighbouring multipliers: for the inverse cascade, $I(\sigma_{i},\sigma_{i+1})≃0.23$, $II(\sigma_{i},\sigma_{i+1},\sigma_{i+2})≃-0.1$. For the direct cascade, $I(\sigma_{i},\sigma_{i+1})≃0.3$, $II((\sigma_{i},\sigma_{i+1},\sigma_{i+2})≃-0.08$. No discernible $I(\sigma_{i},\sigma_{i+k})$ were found for k>1. While $\sigma_{i}$ and $\sigma_{i+2}$ are practically uncorrelated, there is some small synergy in a triplet. For comparison, we find analytically from (7.5): $I_{\text{eq}}(\sigma_{i},\sigma_{i+1})=\ln2-1/2\approx 0.19$.

## 8. Extensivity of entropy and relation between amplitudes and multipliers

The multi-mode entropy $S(a_{1},\ldots ,a_{N})$ is bounded from *above* by the sum of the entropies of the individual modes. As one moves along the cascade, the one-mode entropy decreases linearly in the inertial interval, as seen in figures 2 and 3 (such a decrease will eventually be restricted by a finite resolution). About extra 20% to the decay rate of the entropy per mode comes from the growth of the mutual information between complex amplitudes. Multi-mode correlations must further slow down the growth of the total entropy with the number of degrees of freedom. This shows that the entropy of the statistical distribution of the amplitudes grows with the number of modes slower than linearly, that is the total entropy of the amplitudes must be non-extensive in turbulence, as opposite to thermal equilibrium. Is there any universal ‘area law of turbulence’, like for the entropy of black holes which is proportional to the surface area rather than to the volume? We are unable yet to answer the first question analytically, nor it is feasible to compute numerically the entropy of multidimensional distribution beyond 3–4 modes. The next natural question is whether the entropy of turbulence is sub-extensive in any encoding. The negative answer is given by the Kolmogorov multipliers, which have universal statistics independent of i, as shown above. One consequence of the scale invariance (translation invariance with respect to the mode number) of the statistics of the multipliers is that the respective entropy is extensive, that is proportional to the number of modes. Generally, the entropy depends on the representation or encoding. From the information theory viewpoint, the Kolmogorov multipliers realize representation by (almost) independent components, that is allow for maximal entropy. In other words, computing or measuring turbulence in terms of multipliers gives maximal information per measurement (the absolute maximum is achieved by using the flat distribution, that is the variable u(σ) defined by du=P(σ) dσ).

The amplitudes are expressed via the multipliers

$$X_{k}\equiv \ln x_{k}=\ln\frac{|a_{k}|}{\sqrt{n_{k}}}=\ln x_{M}+\sum_{i=M+1}^{M+k}\sigma_{i}+\frac{1}{2}\log\frac{n_{M}}{n_{k}}.$$

The first term is due to the pumping-connected mode, which correlates weakly with $\sigma_{i}$ in the inertial interval. As was shown above, the correlation between multipliers decays fast with the distance between them. This suggests that the statistics of the amplitude logarithm at large k must have asymptotically a large-deviation form

8.1 $$\ln P(X_{k})=-kH\left(\frac{X_{k}}{k}\right).$$

**Figure 6.**
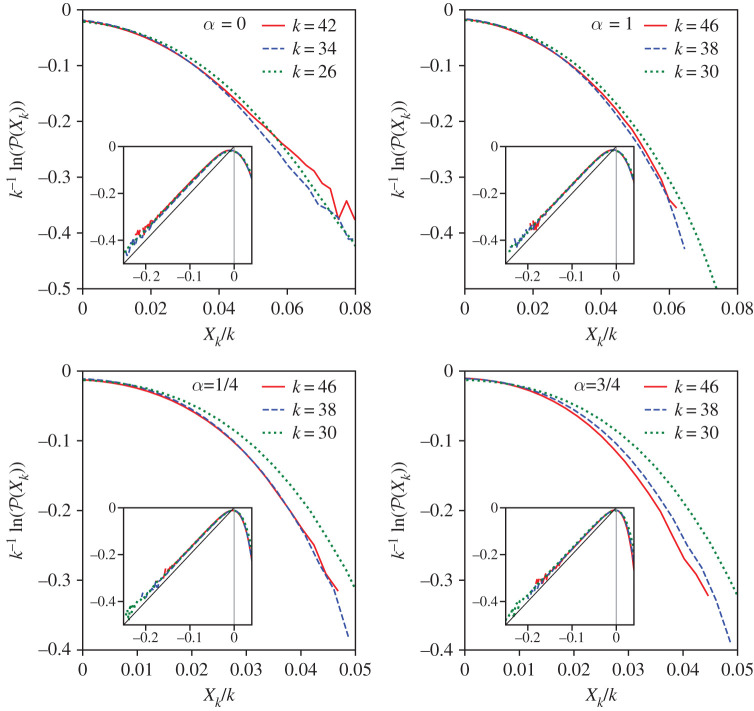
The right parts of the probability distributions of $X=\ln|a_{k}|$ and insets for the whole distributions for inverse (α=0,1/4) and direct (α=3/4,1) cascades. The left slope −2 is due to a simple fact that the moments $\left\langle |a{|}^{q}\rangle =\langle \exp(qX)\right\rangle$ diverge for q≤−2. The distributions collapse to the large-deviation form away from the pumping. (Online version in colour.)

Indeed, sufficiently far from the pumping, the probability distributions collapse in these variables, as shown in figure 6. From the viewpoint of the general multi-fractal form (6.1), such self-similarity means that $g(x_{k}/\lambda^{kh})=g({\text{e}}^{X_{k}-kh\ln\lambda })$ is such a sharp function that the integral in (6.1) is determined by the single $X_{k}$-dependent value, $h(X_{k})=X_{k}/k\ln\lambda$. We then identify f=−H/ln⁡λ.

One can express

8.2 $$\begin{array}{rl} P(X_{k}) & \;=\int \text{d}\sigma_{1}\ldots \text{d}\sigma_{k}P(\sigma_{1},\ldots ,\sigma_{k})\delta \left(X_{k}-\sum_{i=1}^{k}\sigma_{i}\right)\\ & \;=\int_{-\infty }^{\infty }\text{d}y\;{\text{e}}^{-ıyX_{k}}\int \text{d}\sigma_{1}\ldots \text{d}\sigma_{k}P(\sigma_{1},\ldots ,\sigma_{k})\exp\left(ıy\sum_{i=1}^{k}\sigma_{i}\right)\\ & \;\equiv \int_{-\infty }^{\infty }\text{d}y\;{\text{e}}^{-ıyX_{k}}G(y). \end{array}$$

The asymptotes of $P(X_{k})$ are determined by the singularities of the generating function G(y). For $X_{k}\to -\infty$, the leading singularity in the upper half plane is y=2ı. This follows from the fact that for any i there is a finite probability of zero amplitude, so that the moments $\langle |a_{i}{|}^{q}$ diverge for q≤−2. At the left tail, it gives $\lim_{X_{k}\to -\infty }P(X_{k})\propto \exp(-2X_{k})$, which is indeed observed in figure 6. It is not clear at the moment how to find the singularities in the lower half-plane that determine the positive moments and the anomalous exponents.

Of course, if multipliers were statistically independent, then (8.2) would break into a product and one would be able to find the statistics of amplitudes using the standard tools of large deviation theory. Such theories of multiplicative cascades go back to Kolmogorov’s work on ore pulverization, where at every step of the cascade a stone is broken into random pieces independent of the previous steps; assuming a finite variance of multipliers one obtains lognormal distribution of stone sizes [34]. Applying this to fluid turbulence [3] leads to unphysical results, as was noticed by Frisch [1]. Indeed, a lognormal distribution leads to negative $\zeta_{q}$ for sufficiently large q, which does not make sense. In addition, if the multipliers were statistically independent, one would compute ln⁡P(X)=−kH(X/k) or $\zeta_{q}$ proceeding from P(σ) by a standard large-deviation formalism: $H(y)=\min_{z}[zy-G_{0}(z)]$, where $G_{0}(z)=\ln\int \text{d}\sigma \;{\text{e}}^{z\sigma }P(\sigma )$. Such derivation would express $\left\langle |a_{k}{|}^{q}\right\rangle$ via $\left\langle {\text{e}}^{q\sigma_{k}}\right\rangle$, which is impossible since the former moments exist for all q, while the latter do not because of the exponential tails of P(σ), see also [12,13]. Therefore, to describe properly the scaling of the amplitudes, one needs to account for the correlations between multipliers. Physically, it is quite natural that the rule how the distribution changes along the cascade must be encoded in the correlations between the steps of the cascade. The correlation functions are shown in figure 5. The main feature in both cascades is the anti-correlation of the nearest neighbours, as in anti-ferromagnetic. The difference is that the second neighbours are correlated in the inverse cascade and anti-correlated in the direct one. For larger distances, the interaction is again anti-ferromagnetic, that is oscillating at every step and exponentially decaying with the inter-mode distance.

The existence of the self-similar probability distribution for the logarithms of the amplitudes motivates one to look for a non-trivial fixed point of some RG, which sums and re-scales the multipliers. Any traditional RG of the single-time distribution—for instance, hierarchical model [35]—would in our case give a Gaussian $P(X_{k})$, since the variance of σ is finite and the pair correlations decays exponentially. Yet our $P(X_{k})$, is not Gaussian and $P(a_{k})$ is not lognormal. Therefore, our case most likely requires a new approach capable to describe non-equilibrium, like dynamical RG. We need to renormalize the flux of probability or a similar quantity, rather than the single-time pdf. It is also worth bearing in mind that only the single-time statistics of $\sigma_{k}$ is independent of k; fluctuations happen on timescales depending on k. Any attempt to develop non-equilibrium RG to derive $H(X_{k}/k)$ must include time renormalization as well.

One may hope that the asymptotic distribution defined by H(X/k) in the limit of large k is not very sensitive to the particulars of $P(\sigma_{1},\ldots ,\sigma_{k})$. Yet it would be very interesting to see how a weak correlations between neighbouring multipliers make all moments of the amplitude finite, overcoming divergency due to exponential tails of P(σ). While the body and the (trivial) left tail of H(X/k) can be computed neglecting weak correlations between the multipliers, the positive moments $\left\langle |a{|}^{q}\rangle =\int \text{d}X\exp[Xq-kH(X/k)\right]$ are determined by the far right tail of H, which must be sensitive to the correlations.

## 9. Conclusion

This note weaves together three threads introduced by Uriel Frisch into turbulence studies: criterion for cascade direction, breakdown of self-similarity, and multi-fractal formalism. The universal family of models (2.7) introduced here provides for a unique possibility to use perturbative approach in a system with strong interaction. That possibility is based on the remarkable fact that thermal equilibrium has exactly Gaussian statistics with independent modes; varying the parameter of our family, one is able to study turbulence close to thermal equilibrium.

For our models or velocities in hydrodynamics, the results shed light on what is crucial for the breakdown of scale invariance in the statistics of amplitudes. The statistics deviates from Gaussian as one goes into the cascade, as long as the interaction time decreases, regardless whether the cascade is direct or inverse.

Shell models in general and our family in particular demonstrate that turbulence is qualitatively different from ore pulverization [34]. In the latter case, the assumption of the statistical independence of multipliers makes sense. In turbulence, not only multipliers are correlated but their equations of motion are explicitly not translation invariant, while the equations on the amplitudes are. And yet, the amplitudes do not have the translation-invariant statistics, while the multipliers do. Far from the pumping, multipliers acquire universal statistics, which determines, in particular, the anomalous scaling exponents of the amplitudes or, equivalently, the way the statistics of the amplitudes changes along the cascade.

## Data accessibility

This article has no additional data.
